# Neuregulin Upregulates Microglial α7 Nicotinic Acetylcholine Receptor Expression in Immortalized Cell Lines: Implications for Regulating Neuroinflammation

**DOI:** 10.1371/journal.pone.0070338

**Published:** 2013-07-30

**Authors:** Malwina Mencel, Michelle Nash, Christian Jacobson

**Affiliations:** 1 Department of Biology, University of Waterloo, Waterloo, Ontario, Canada; 2 National School of Medicine, University of Namibia, Pionierspark, Windhoek, Namibia; Massachusetts General Hospital and Harvard Medical School, United States of America

## Abstract

Neuregulin, previously known as ARIA, is a signaling protein involved in cell survival, synaptic plasticity, cell communication and differentiation. Neuregulin has also been described as a potent inducer of acetylcholine receptor transcription in muscle and although both neuregulin and acetylcholine have been individually described to have neuroprotective roles, their relationship in the cholinergic anti-inflammatory pathway of the brain has not been examined.

Using three cell lines, BV-2, EOC-20 and RAW 264.7, we investigated the role that neuregulin signaling through the Erb family of tyrosine kinases may play in the anti-inflammatory process mediated by the α7 nicotinic acetylcholine receptors. Here we show that ErbB4 is expressed in all of our cell lines and is phosphorylated upon treatment with neuregulin. Neuregulin treatment further increases the expression of α7 nicotinic acetylcholine receptors in the microglial lines tested. Given the central role of α7 nicotinic acetylcholine receptors in regulating system inflammation we analyzed the expression of several pro-inflammatory cytokines in our system. Using ELISAs for TNF-α and IL-6 we show that treatment with NRG can produce a nearly a 33% decrease in the levels of tumor necrosis factor-α secreted by activated microglia and a nearly 88% decrease in IL-6.

Given these results we propose a neuroprotective role for neuregulin wherein it modulates the expression of TNF-α and thus inflammation in the CNS via the upregulation of α7 nicotinic acetylcholine receptor expression in microglia in vitro. We suggest that the disregulation of neuregulin expression may be pivotal in neurological disorders characterized by inflammation.

## Introduction

Inflammation is an acute innate immune response to pathogens and functions to eradicate infection by phagocytosis and the recruitment of other effector cells via the rapid production of cytokines such as tumor necrosis factor (TNF)-α, interleukin (IL)-1β, IL-6, chemokines and other pro-inflammatory mediators, by resident tissue macrophages [1–5]. If this local response becomes over-whelmed by the number of pathogens and/or the amount of pro-inflammatory signals becomes excessive, the response can turn systemic resulting in sepsis, organ failure and even death. Innate deregulation alone can lead to a variety of auto-inflammatory diseases such as arthritis, asthma and Crohn’s disease [6–8].

The central nervous system (CNS) is an immunologically privileged site that is inaccessible to cells of the systemic immune system due to the blood brain barrier (BBB). Microglia represent the macrophages of the CNS innate immune system and comprise 10-12% of the total cell population of the brain [9,10]. Microglia are activated under typical pathological conditions and can act as specialized sensors of brain tissue injury. Activated microglia synthesize neuroinflammatory molecules; the synthesis of which correlates with a variety of neurodegenerative diseases, stroke, traumatic injury and tumor invasion [10,11]. For instance, the preliminary stages of Alzheimer’s disease (AD) progression contain inflammatory components; a result of microglial activation by functional peptides e.g. amyloid beta (Aβ) [3,9,12,13].

In the periphery, macrophages are able to aid in the inhibition of systemic outcomes via a neuro-immune axis termed the cholinergic anti-inflammatory pathway [14]. Acetylcholine (ACh), secreted by the vagus nerve in response to elevated levels of pro-inflammatory mediators and bacterial products, such as lipopolysaccharide (LPS), binds to homopentameric α7 nicotinic acetylcholine receptors (α7nAChRs) expressed by macrophages [1,14]. This prevents further synthesis of pro-inflammatory cytokines, especially TNF-α, inhibiting the pro-inflammatory feedback loop [8]. However, this pathway does not affect the synthesis of the homeostatic anti-inflammatory cytokine IL-10 [8]. A similar cholinergic mechanism has also been demonstrated in the CNS by microglia [15]. Finding a pathway that regulates the transcription of α7nAChR in microglia could potentially lead to a mechanism for regulating the inflammatory response in the CNS.

The neuregulin (NRG) family of ligands are important in cell-cell communication in development and disease and are known to play roles in synaptogenesis and neuronal survival [16]. NRGs all contain an epidermal growth factor (EGF) domain and are extensively alternatively spliced to produce various isoforms [17]. It is through the EGF domain that the various NRG isoforms interact with ErbB family of transmembrane tyrosine kinases receptors [17].

The ErbB family of receptors is comprised of four members: EGFR, ErbB2, ErbB3 and ErbB4. These receptors are expressed in various cell and tissue types with NRG1 binding principally, and with highest affinity, to ErbB3 or ErbB4 [18]. NRG binding to the Erbs induces the formation of functional ErbB homo-and/or heterodimers. Ultimately, these interactions result in receptor phosphotyrosylation and the activation of intracellular signaling cascades. At the neuromuscular junction (NMJ), NRG is involved in the induction of a signal transduction cascade that results in the activation of AChR subunit gene transcription [19]. If NRG is able to regulate AChR in muscle cells, perhaps it is possible that a similar mechanism resulting in regulation of AChR expression exists in microglial cells.

Here we investigated the possible effects NRG1 may have on microglia. In particular we were interested in the possible up-regulation of α7nAChR expression and the potential for NRG1 and its receptors, the ErbBs to regulate inflammatory responses. Initial experiments examined ErbB receptor expression in several microglial cell lines. Immunoblots revealed that ErbB4 is the predominat ErbB isoform expressed in microglia and that these receptors were phosphotyrosylated in response to NRG1 treatment. Having established that microglia express functional ErbB4 we turned our attention to the expression of α7nAChRs. In the BV-2 microglial cell line we found a significant increase in α7nAChRs with NRG1 treatment compared to control cultures. To determine if, as in peripheral macrophages, increased α7nAChR expression had any effect on inflammatory responses [1] we then assayed NRG1 treated and LPS induced microglia for changes in TNF-α and IL-6 expression with and without ACh. Since TNF-α and IL-6 are pro-inflammatory cytokines, a decrease in expression is typically associated with a reduced immune response. Using a commercially available enzyme-linked immunosorbent assay (ELISA) we found that in cells pre-treated with ACh and immunologically challenged with LPS, NRG does appear to induce a decrease in TNF-α and IL-6. Collectively, these results suggest a role for NRG working in concert with ACh to decrease inflammation. This has potentially interesting implications in Alzheimer’s disease and needs to be investigated further.

## Materials and Methods

### Cell Lines and Culture

The EOC-20 murine microglial cell line (CRL-2469; ATCC) was cultured in Dulbecco’s modified Eagle’s medium (DMEM; Wisent, St. Bruno, QC) with 4mM L-glutamine supplemented with 10% fetal bovine serum (FBS; Wisent), 20% LADMAC conditioned media containing colony stimulating factor (CSF)-1, 100IU/mL penicillin and 100mg/mL streptomycin (Wisent). LADMACs, a murine lymphoblast cell line (CRL-2420; ATCC), were cultured for the production of CSF-1 in Eagle’s Minimum Essential medium (EMEM; Wisent) supplemented with 10% FBS, 100IU/mL penicillin and 100mg/mL streptomycin. Confluent LADMACs were not passaged for 5 to 7 days to allow for an accumulation of secreted CSF-1 in the media. The LADMAC conditioned media was then filter sterilized using a 0.2µM pore size filter, aliquoted, frozen and stored at -20 degrees Celsius for future use. The BV-2 murine microglial cell line (received as a gift from Dr. Michael J. Strong, University of Western Ontario, London, ON) is an immortalized cell line that demonstrates the functional and morphological characteristics of microglia [20,21]. BV-2s and the murine macrophage cell line RAW264.7 (TIB-71; ATCC) were cultured in RPMI 1640 (Wisent) supplemented with 10% heat inactivated FBS, 100IU/mL penicillin and 100mg/mL streptomycin. HeLa S3 cells, derived from a human cervical epithelial cell line (CCl-2.2; ATCC), were cultured in the same manner as BV-2 and RAW264.7 cell lines. The C2C12 murine myoblast cell line (CRL-1772; ATCC) was cultured in low glucose DMEM (Wisent) supplemented with 10% FBS, 100IU/mL penicillin and 100mg/mL streptomycin. When cells reached 80% confluency, culture media was changed to DMEM supplemented with 2% horse serum (Wisent), 100IU/mL penicillin and 100mg/mL streptomycin to allow for the differentiation of myoblasts to myotubes. All cell lines were grown at 37 degrees Celsius and 5% CO_2_ with the exception of C2C12 cells which were grown at 8% CO_2_.

### Cell Culture Treatment Conditions

Cells growth media was refreshed prior to treatments. Cells were either treated with 1nM rh-NRG-1 (Shenandoah Biotechnology, Warwick, PA), here on in referred to as NRG, over night, 100ng/ml LPS for 4 hours, both NRG (Shenandoah Biotechnology) and LPS (Sigma-Aldrich, St. Louis, MO), 1mg/ml bovine serum albumin (BSA; Wisent) overnight, or left untreated, unless otherwise stated.

### Cell Lysis and Protein Extraction

Cells were briefly washed twice in cold Dulbecco’s phosphate buffer saline (D-PBS; Wisent) and harvested with a rubber policeman in D–PBS. Cells were then spun at 1500 rpm for 10 minutes at 4 degrees Celsius to pellet and supernatants were discarded. Cell pellets were re-suspended and lysed with protein extraction buffer [25mM Tris pH 7.5, 25mM Glycine, 150mM NaCl, 1X Complete Protease Inhibitor Cocktail (Roche, Mississauga, ON), 1% Triton X-100 (Sigma-Aldrich) and 5mM EDTA pH 8.0 (Quality Biological Inc., Gaithersburg, MD)]. Cell resuspension was incubated on ice for 15 minutes and then centrifuged at 14000 rpm for 5 minutes at 4 degrees Celsius. Pellets containing cell debris were discarded. Protein extractions completed for phosphotyrosine experiments included the addition 1mM sodium orthovanadate (Sigma-Aldrich) to D–PBS and to protein extraction buffers.

### Immunoprecipitation of ErbB receptors and α7nAChRs

ErbB receptors were immunoprecipitated from protein samples by incubation with rabbit polyclonal anti-ErbB-2 (SC-284; Santa Cruz Biotechnologies, Santa Cruz, CA), 3 (SC-285; Santa Cruz), or 4 (SC-283; Santa Cruz) antibodies for 1 hour at 4 degrees Celsius with gentle rocking. Subsequently, protein G agarose beads (Millipore, Billerica, MA) were added to each sample to pull-down the antibody-antigen complex and incubated an additional hour at 4 degrees Celsius. Similairly, α7nAChRs were immunoprecipitated from protein extracts by incubation with biotin-XX-α-bungarotoxin (Biotium Inc., Burlington, ON), which binds to α1, α7 and α9 subunits of the nAChR family, at 4 degrees celsius for 1 hour with gentle rocking. Samples were then incubated for an additional hour with streptavidin-sepharose beads (Upstate Biotechnology, Lake Placid, NY) at 4 degrees Celsius with gentle rocking. Beads were washed and reserved for western blot analysis.

### Detection of ErbB Receptors and α7nAChRs by Western Blotting

ErbB2-4 and α7nAChR expression in microglia and macrophages were confirmed by immunoblotting. Samples were resolved by electrophoresis on 8% SDS-polyacrylamide gels and then blotted onto nitrocellulose membranes. Membranes were blocked overnight with 2.5% bovine serum albumin (BSA)/2.5% non-fat dehydrated milk in TBS-T and then incubated with different rabbit anti-ErbB polyclonal antibodies (Santa Cruz) or rabbit anti-α7nAChR polyclonal antibody (ab23832; Abcam, Cambridge, MA). Membranes were then incubated with HRP-conjugated donkey-anti-rabbit secondary antibody (Amersham, Arlington Heights, IL). Immunoreactivity was detected using ECL (Pierce, Rockford, IL) and captured on autoradiography film (GE, Mississauga, ON). Molecular weight markers were used to determine protein size (Fermentas, Burlington, ON). C2C12 and HeLa protein extracts were used for ErbB2-3 [22] and ErbB4 [23] positive controls, respectively. Anti-mouse β-tubulin (a gift from Dr. Mungo Marsden, University of Waterloo, Waterloo, ON), used to ensure equal protein loading, was detected with rabbit anti-mouse HRP (Amersham). Crude protein extracts were resolved by electrophoresis as above for β-tubulin immunoblots and in parallel with ErbB receptor immunoblots. For semi-quantitative analysis, the relative intensity of protein bands was determined by densitometry using NIH ImageJ software (NIH, Bethesda, MD).

### Detection of ErbB Receptor Phosphotyrosine Activity by Western Blotting

Phosphotyrosine activity of different ErbB receptors was detected by immunoblotting. Samples were resolved by electrophoresis, blotted onto nitrocellulose, as indicated previously, and then blocked in 5% BSA. Membranes were incubated with 4G10 monoclonal anti-phosphotyrosine antibody (Upstate) and then visualized by incubating with rabbit anti-mouse HRP (Amersham) and detecting with ECL (Pierce).

### Analysis of secreted TNF-α and IL-6 by ELISA

The concentration of secreted TNF-α and IL-6 in cell culture media was determined by ELISA following the manufacturer’s protocol (88-7324 and 88-7064, respectively; eBioscience, San Diego, CA). Cell culture media harvested from RAW264.7 and BV-2 cells post-treatments was stored at -80 degrees Celsius until needed. RAW264.7 cells were treated as mentioned previously. BV-2 cells were either pre-treated for 30 minutes with 1mM pyridostigmine bromide (Sigma-Aldrich), an acetylcholinesterase inhibitor, and 100µM acetylcholine chloride (ACh; Sigma-Aldrich) or left untreated (adapted from [14], [1] and [15]). Then all BV-2 cells were treated for 4 hours with either 1nM NRG (Shenandoah Biotechnology), 100ng/ml LPS (Sigma-Aldrich), both NRG (Shenandoah Biotechnology) and LPS (Sigma-Aldrich), or left untreated.

### Statistical Analyses

Measurements are expressed as means ± SEM. Statistical analysis was performed using Student’s *t*-test to compare two groups. Statistical significance was designated as *p*<0.05 or *p*<0.01, as stated. Statistical significance of differences between groups was analyzed by ANOVA with Tukey’s Post-Hoc test for multiple comparisons, where indicated. Differences were considered significant when *P* values were less that 0.05, using Kaleidagraph 4.1.1 (Synergy Software, Reading, PA).

## Results

### ErbB4 receptors are expressed in microglia and macrophages

**Figure 1:**
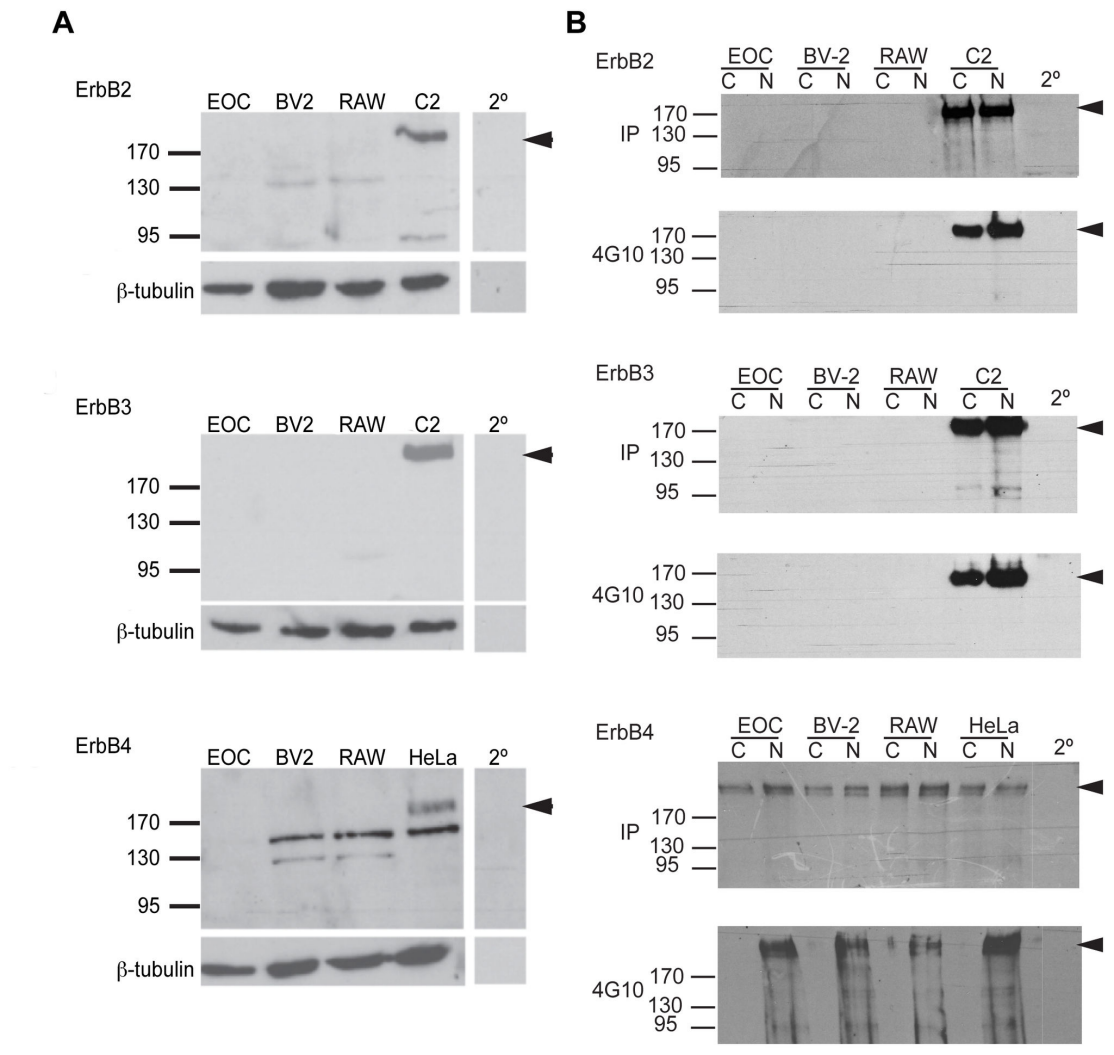
ErbB2-4 receptor expression and phosphotyrosine activity in microglial and macrophage cell lines. Immunoblots of whole cell lysates (panel A) or isolates from immunoprecipitations using anti-ErbB2, anti-ErbB3 and anti-ErbB4 antibodies (panel B) from microglial cell lines (EOC and BV-2) and macrophage (RAW) were probed with antibodies specific for each ErbB receptor. Arrows to the right of each panel indicate the position of each expected immunoreactive species and its mass (185 kDa). Whole lysates from EOC-20, BV-2 and RAW264.7 cell lines immunoblotted with the anti-ErbB2, anti-ErbB3 and anti-ErbB4 antibodies showed no detectable levels of these proteins in each of these cell line (A). However, immunoprecipitated samples from the same cell lines indicate that ErbB4 is expressed (IP, bottom panel B) and that ErbB4 phosphotyrosylation in these cells increased with neuregulin treatment (4G10, bottom panel B). Positive control cell lines were C2C12 for ErbB2 and 3 and HeLa S3 (H) for ErbB4. Secondary controls (shown at right) were probed with secondary antibody only. Mouse anti-β-tubulin antibody indicates equal loading of cell extracts indicated by the band at the bottom of each ErbB panel at 50kDa. Lack of detectable banding in EOC, BV-2 and RAW may indicate no or low levels of the ErbB receptors.

NRG1 binds to ErbB family of tyrosine kinase receptors and initiates intracellular signalling resulting in significant cellular responses including cell proliferation, survival and migration [16]. Working with a number of immortalized glial and macrophage cells lines our initial investigations centered on the expression of and possible functionality of the ErbB receptors in these cell lines. Crude, whole cell extracts were prepared from the murine microglial cell lines EOC-20 and BV-2, and the macrophage cell line RAW264.7. Following separation using electrophoresis and transfer to nitrocellulose, membranes were immunoblotted with various anti-ErbB antisera. The ErbB receptors have molecular weights of approximately 185kDa, and specific staining is clearly identifiable in our control lanes that contained crude extracts from the C2C12 and HeLa S3 cell lines, which serve as positive controls for ErbB2-3 and ErbB4 respectively (Figure 1A). In our glial and macrophage lines either the ErbB receptors were not expressed or the level of expression was below the threshold of detection, as can be seen in Figure 1A. Previous findings in primary microglial cell lines suggested that some but not all isoforms of the ErbB receptors maybe expressed at low concentrations [24–26]. In an attempt to concentrate potentially low concentrations of ErbB2-4 receptors that may be present in these cells, we immunoprecipitated individual receptors using the appropriate antisera. Immunoblots of protein extracts immunoprecipitated with ErbB4 antisera demonstrate that the ErbB4 receptor is present in EOC-20, BV-2 and RAW264.7 cell lines (Figure 1B). However, there was no visible banding apparent for immunoprecipitates of ErbB2 and ErbB3 in all three cell lines (Figure 1B). Thus, ErbB4 receptors are present in microglia and macrophages, although at low concentrations.

### NRG induces phosphotyrosine activity of ErbB4 receptors on microglia and macrophages

The ErbB receptors dimerize and become phosphorylated upon ligand binding [27]. Given this, we next investigated whether ErbB4 forms a functional receptor for its ligand, NRG1, in our cell lines. EOC-20, BV-2 and RAW264.7 cell lines were treated with 1nM NRG1 overnight prior to harvest and extraction. Protein extracts were then subjected to immunoprecipitation with antisera specific for ErbB4 receptors. Extracts from NRG treated culture samples were compared to untreated control sample extracts on immunoblots probed with the anti-phosphotyrosine antibody 4G10. NRG treated samples compared to untreated or control samples, from all three cell lines demonstrate NRG induced phosphorylation of the ErbB4 tyrosine kinase compared to untreated controls (Figure 1B). Again we saw no detectable phosphotyrosine activity of ErbB2 and ErbB3. Collectively, these results indicate that ErbB4, likely in the form of a homodimer, can act as a functional receptor for NRG1 in these cell lines.

### NRG addition induces an increase in α7nAChR expression in microglia and macrophages

**Figure 2:**
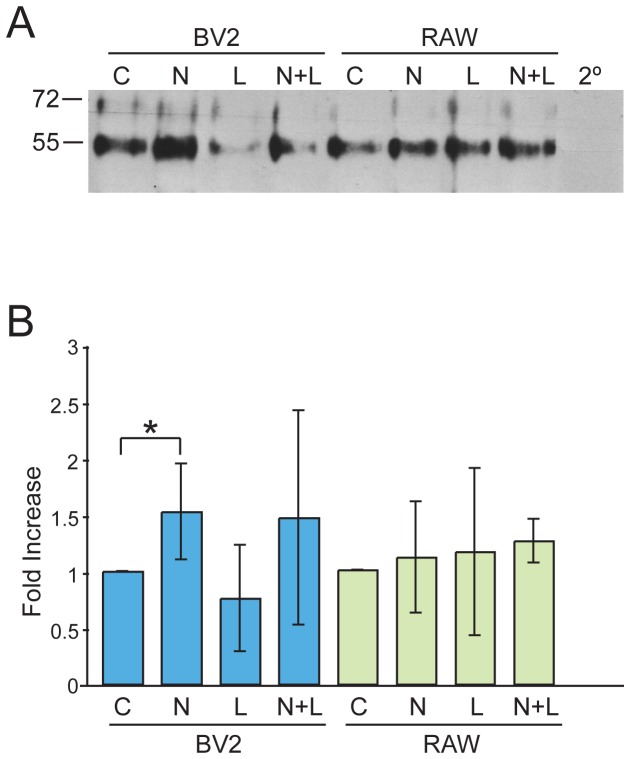
NRG increases α7nAChR expression in microglia and macrophages. Microglia (BV-2) and macrophage (RAW) cell cultures were treated overnight with NRG (N) or left untreated (C). LPS (L) was added to activate cells four hours prior to extraction. Protein extracts were then precipitated with biotin-conjugated α-BTX and streptavidin agarose beads. Panel A is a representative immunoblot probed with anti-α7nAChR antisera. The α7nAChR band is shown at approximately 55kDa. Subsequent densitometric analysis of immunoblots shows a 1.53 ± 0.42 fold increase in α7nAChRs over control in NRG treated BV-2 cells (n=3, *p* <0.05, t-test). BV-2 cells appeared to show a decrease in α7nAChR expression when treated with LPS, 0.76 ± 0.47 compared to untreated cells, however LPS effect on BV-2 cells was variable. RAW264.7 cells treated with NRG showed a 1.11± 0.49 fold increase over untreated cells. Taken together our results indicate that NRG increases α7nAChR expression in BV-2 cells but have little effect in RAW264.7 cells. Values are means ± SEM. Error bars represent SEM.

In muscle, functional homodimerization of ErbB4 upon binding of NRG can regulate the transcription of acetylcholine receptors [27]. We investigated NRG1 function on α7nAChR expression in microglial and macrophage cell lines to determine if NRG1 has a similar inductive effect on the expression of α7nAChRs. BV-2 and RAW264.7 cell lines were treated with 1nM of NRG1 in culture overnight prior to extraction. Since LPS activates inflammatory responses in microglia and macrophages, additional treatment conditions for BV-2 and RAW264.7 were included; LPS and LPS in addition to NRG compared to untreated. Using protein extracted from cultures that have undergone the aforementioned treatments, α7nAChRs were pulled down using biotin-conjugated α-bungarotoxin and probed using α7nAChR antisera. Immunoblots for α7nAChRs were examined for changes in protein expression levels as a result of treatment conditions compared to controls and then were analyzed using semi-quantitative densitometry. Figure 2 shows a representative western blot of α7nAChR. In RAW264.7 cells we saw no significant changes in α7nAChR expression in treated versus untreated cells (Figure 2B). However, BV-2 cells treated with NRG for over 24 hours showed a marked increase in α7nAChR expression of approximately 1.5±0.42 fold increase (n=3, *p*<0.05, Student’s t-test; Figure 2B). Unconjugated α-bungarotoxin added to cultures prior to treatment was capable of blocking this effect (data not shown). Though use of commercial antibodies against the α7 subunit has undergone some scrutiny in the past for lack of specificity, we have selected antibodies that have been previously confirmed to be specific for α7nAChR [28].

### NRG addition results in a decrease of the pro-inflammatory cytokine TNF-α in the presence ACh in microglia

**Figure 3:**
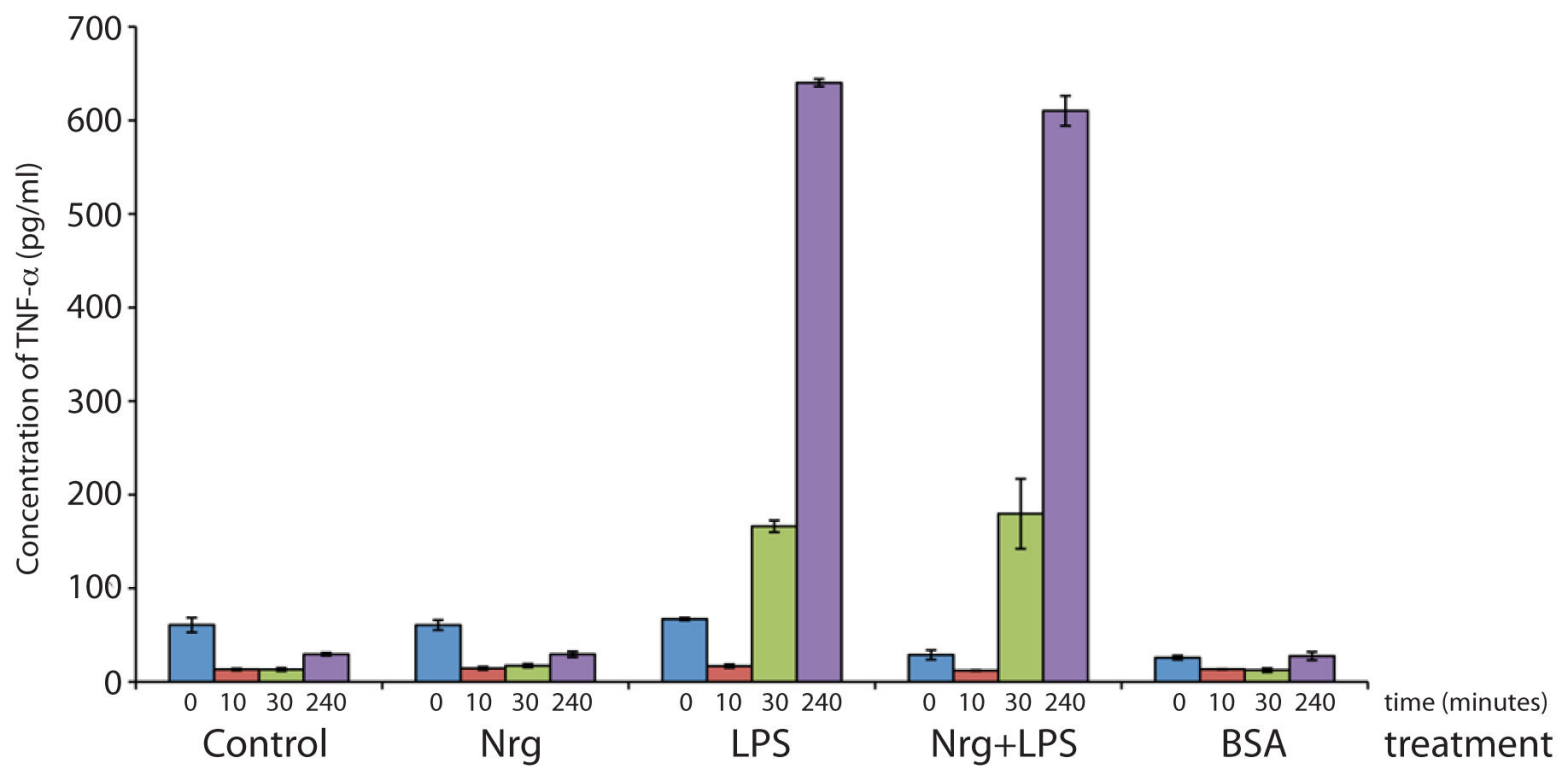
NRG does not affect TNF-α synthesis in macrophages. RAW 264.7 cells were challenged with LPS and the resulting induction of TNF-α determined at several time points. TNF-α concentrations were determined using cell culture media post-treatments by a commercially available ELISA kit. An additional treatment completed with BSA was added as a control for the experiment. Here we show that NRG addition 24 hours prior to LPS did not have an effect on TNF-α concentration in culture media, 610±15.98pg/mL, compared to LPS alone, 640±4.04pg/mL. These results suggest that, at least acutely, NRG alone does not induce a change in TNF-α synthesis.

The α7nAChRs have been reported to regulate inflammation via a cholinergic anti-inflammatory pathway in microglia and macrophages [1,15]. Also, potential neuroprotective roles for both NRG1 [26] and α7nAChRs [29] have been previously reported, and the progress of AD has been described as a function of neuroinflammation [10]. As such, we subsequently investigated the effects of NRG1 on the synthesis of one of the major key mediators of inflammation, TNF-α. Using a commercially available TNF-α ELISA kit, media harvested from treated and untreated cells in culture were analyzed for secreted TNF-α. RAW264.7 cells were initially used as macrophage model to determine if treatment with NRG1 could offer protection from challenge with LPS in vitro (Figure 3). RAW264.7 cells were subjected to treatment with NRG (24 hours), LPS (4 hours) or NRG+LPS together and culture medium was harvested at 10 minutes, 30 minutes and 240 minutes time-points after treatments. A BSA treatment was added as an additional control to identify unintentional activation of macrophages (Figure 3) and microglia (not shown). Figure 3 shows that NRG addition alone four hours prior to LPS treatment does not have an effect on the concentration of TNF-α (610 ±15.98 pg/mL) compared to LPS alone (640 ±4.04 pg/mL, n=3). However, in LPS treated cells the concentration of TNF-α increased compared to untreated cells (29.47±1.58 pg/mL, n=3). Therefore in RAW264.7 cells, NRG does not have an affect on α7nAChR (Figure 2) or on TNF-α synthesis (Figure 3). We were particularly interested if NRG would act differently on our BV-2 microglial cells.

**Figure 4:**
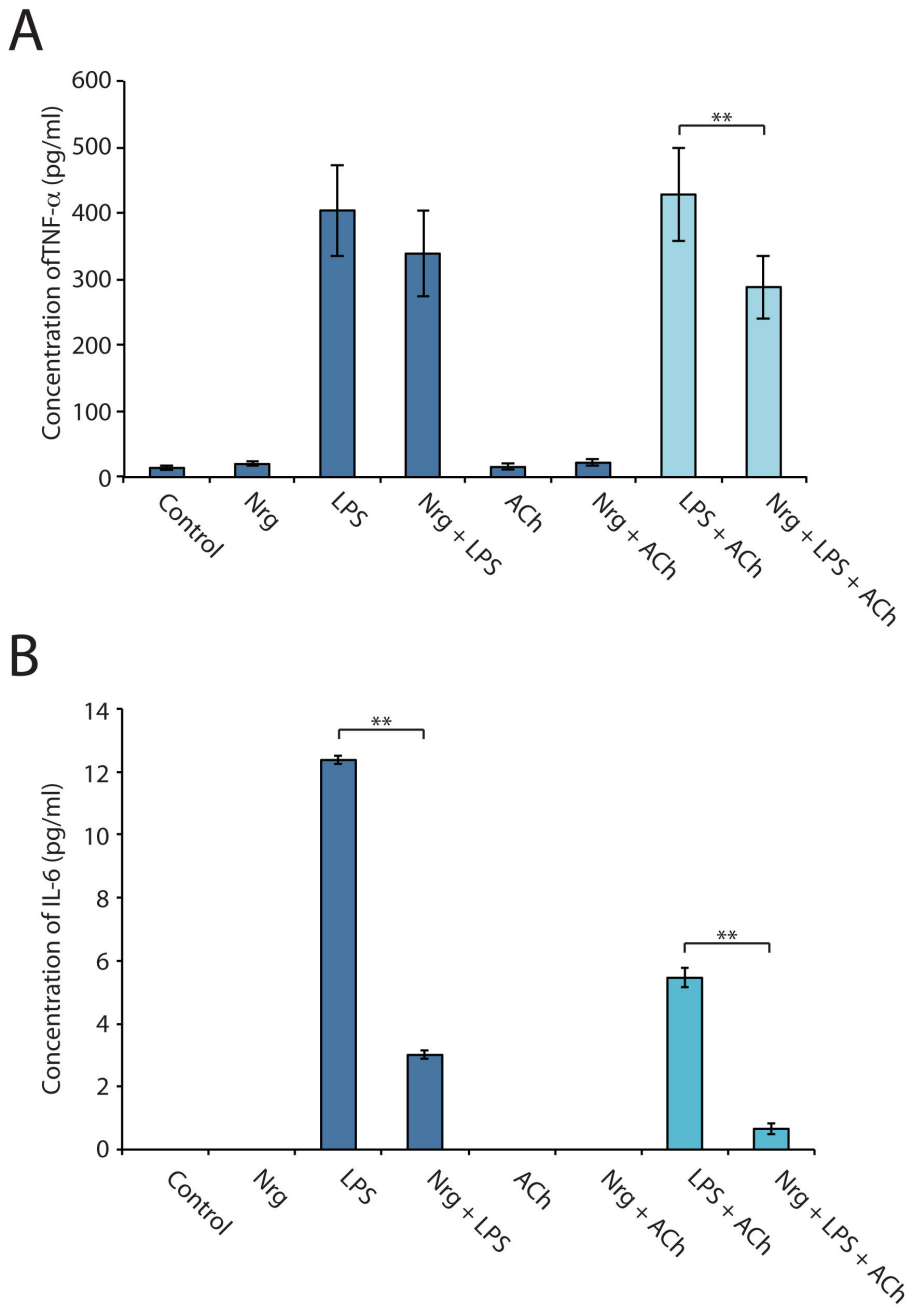
NRG in the presence of ACh induces a significant decrease in secreted TNF-α and IL-6 levels in LPS challenged microglia. Media from untreated (control), NRG, LPS, or both NRG and LPS, in the presence or absence of ACh was analyzed for TNF-α and IL-6 concentrations using a commercially available ELISA kit. BV-2 cells pretreated with ACh and then treated with LPS showed a concentration of 428.02±70.24pg/mL in TNF-α compared to untreated cells while BV-2 cells pretreated with ACh then treated with NRG and LPS showed a concentration of 287.80±47.69pg/mL (n=7, *p*<0.01, Student’s t-test). Interlukin-6 levels significantly decreased with neuregulin treatment with (0.67±0.17pg/mL, n=3, *p*<0.0001, ANOVA) or without ACh (3.01±0.12pg/mL, n=3, *p*<0.05). Taken together our results indicate that BV-2 cells in the presence of ACh and NRG and immunologically challenged with LPS result in a 32.76% decrease in TNF-α and a 87.73% decrease in IL-6 compared to BV-2 cells pretreated with ACh and challenged with LPS alone.

Since ACh is known to interact with the α7nAChRs and thus inhibit pro-inflammatory cytokine synthesis, additional treatment conditions were included where in pre-treatment with ACh for 30 minutes for each condition was examined. BV-2 treatment durations for NRG and LPS conditions were adjusted to 4 hours in culture in order to mimic a more realistic physiological model for innate immune responses. Similar to RAW264.7 cells, LPS addition in BV-2 cells resulted in an increase in the concentration of TNF-α to 403.87±69.10pg/mL compared to untreated cells at 13.87±3.01pg/mL (n=7; Figure 4A). The concentration of TNF-α also increased with NRG and LPS treatments over untreated cells, however this combination did not significantly affect TNF-α concentration (338.48±64.64pg/mL, n=7; Figure 4A) compared to LPS alone. It was the addition of ACh that produced a dramatic decease in TNF-α in cells also treated with NRG and LPS. ELISA analysis showed a 32.76% decrease in TNF-α between cells treated with ACh, NRG and LPS, 287.80±47.69pg/mL, compared to ACh and LPS alone 428.02±70.24pg/mL (n=7, *p*<0.01, Student’s t-test; Figure 4A).

NRG addition modulates the expression of the pro-inflammatory cytokine IL-6, but not anti-inflammatory IL-10 in the presence or absence of ACh in microglia

In addition to TNF-α, the pro-inflammatory cytokine IL-6 has also been implicated as a major player in inflammatory responses to IL-6. In addition, it is yet another factor modulated by the α7nAChR of the cholinergic anti-inflammatory pathway. For this reason we further investigated the effects of NRG by analyzing IL-6 synthesis in the BV-2 cell line. Using a commercially available IL-6 ELISA kit, media harvested from treated and untreated cells in culture were analyzed for secreted IL-6 in the same manner as for TNF-α (Figure 4B). Similar to TNF-α, LPS addition in BV-2 cells resulted in an increase in the concentration of IL-6 to 12.37±0.12pg/mL compared to untreated cells at 0pg/mL (n=3, *p*<0.0001 ANOVA; Figure 4B). The concentration of IL-6 also increased with NRG and LPS treatments over untreated cells, however, unlike for TNF-α, this combination did affect IL-6 concentration (3.01±0.12pg/mL, n=3; Figure 4B) compared to LPS alone. NRG addition alone resulted in a 75.67% decrease in IL-6 compared to LPS (n=3, *p*<0.05, Student’s t-test; Figure 4B). Furthermore, the addition of ACh produced a decrease in IL-6 in cells also treated with NRG and LPS. ELISA analysis showed an 87.73% decrease in IL-6 between cells treated with ACh, NRG and LPS, 0.67±0.17pg/mL, compared to ACh and LPS alone 5.46±0.30pg/mL (n=3; Figure 4B). Comparatively, the concentration of IL-6 is low versus TNF-α, but undergoes a substantially greater relative reduction in expression. Levels of the anti-inflammatory cytokine IL-10 were also examined, however NRG did not appear to induce any changes in IL-10 in the presence or absence of ACh (data not shown).

## Discussion

The AChRs have diverse roles in various tissues. In microglia and macrophages they are important regulators of inflammation. We hypothesized that NRG1, through its binding to the ErbB receptors may increase the transcription of α7nAChRs in microglia. This would have interesting implications in the regulation of inflammation as previous work indicates that increases in α7nAChR expression can lead to a decrease in TNF-α, a known stimulator of inflammation [1,30]. Here we have documented ErbB expression in three immortalized cell lines and shown that NRG1 induces the phosphotyrosylation of the ErbB4 receptor in these cells. Further we have shown that NRG1 treatment can affect an increase in α7nAChR expression. Finally, in appears that NRG1 treatment in conjunction with ACh has a dramatic effect on the secretion of TNF-α in microglial cells.

**Figure 5:**
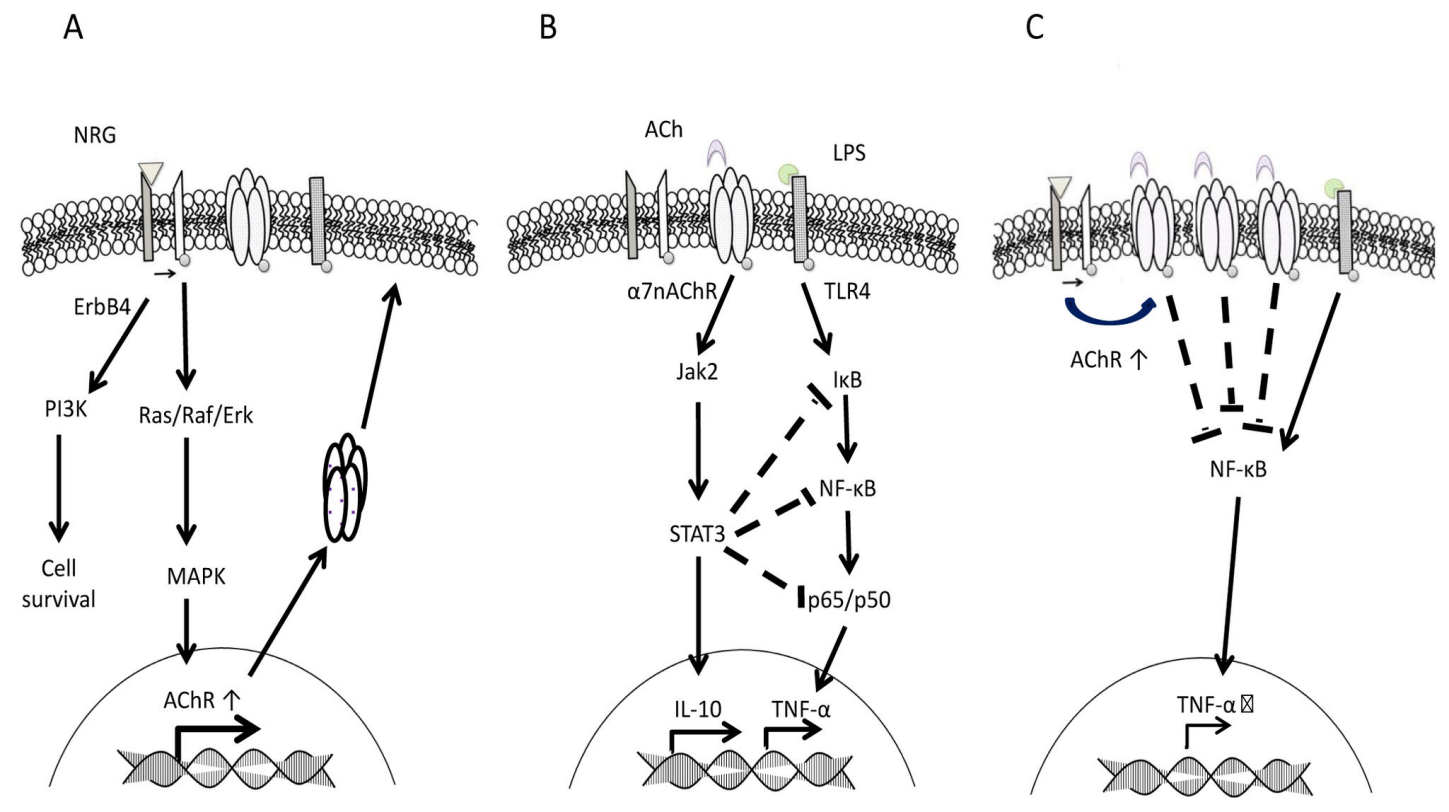
Proposed mechanism of NRGs neuroprotective action during inflammation. Here we propose a model where in NRG amplifies the cholinergic anti-inflammatory pathway mediated by ACh and α7 nAChR during an inflammatory response. In panel A, we describe the possible signaling pathway responsible for the increased expression of α7nAChRs observed in microglia when treated with NRG. NRG signaling through ErbB4 activates Ras/Raf/ERK of the MAPK pathway which leads to cell survival, and in muscle has been shown to activate the N-box response elements associated with the transcription of AChRs. Also of importance to NRGs neuroprotective function is the activation of the p85 subunit of PI3K pathway, its downstream effector PKB/Akt, and Jak/STAT pathways [16,51,52] (not shown); signaling mechanisms also involved in cell survival. In panel B, we describe the well established cholinergic anti-inflammatory pathway [modified from 38]. In response to inflammatory mediators, activation of the α7nAChR by its ligand ACh inhibits the synthesis of TNF-α by preventing the nuclear translocation of the p65 subunit of NF-κB. In addition, the activation of Jak2/STAT3 supports the synthesis of anti-inflammatory cytokine IL-10 and further attenuates TNF-α production by STAT3 action on IκK, NF-κB and p65. In panel C, we propose that in LPS-challenged microglia NRG acting in concert with ACh leads to increased reduction of TNF-α by inducing increased expression of α7nAChR and as such amplifying the cholinergic anti-inflammatory pathway by providing more receptors for ligand binding.

NRG and α7nAChRs have been suggested, individually, to have neuroprotective roles in the brain [26,29], respectively. Here we propose that NRG may support the cholinergic anti-inflammatory pathway by increasing the α7nAChRs present during an inflammatory response and as a result produce microglial mediated neuroprotective response to ACh (see Figure 5 for proposed model). This response may have further implications in hindering the progression of AD, which originates from neuroinflammatory factors and the damage associated therein.

### ErbB4 is expressed by immortalized microglia and macrophages

Although no research has directly investigated ErbB expression in BV-2, RAW or EOC-20 cell lines, a similar immortalized cell line, N9, has been used to investigate ErbB expression. Dimayuga and colleagues (2003) have show that N9 murine microglial cells express ErbB2-4 and are affected by NRG1 addition. They were able to show that the addition of NRG1 was able to reduce the amount of nitric oxide released by microglia in response to an assault (LPS). Nitric oxide release has been found to trigger the expression of pro-inflammatory cytokines such as TNF-α. It is therefore possible that NRG1 may also reduce TNF-α produced by microglia under our conditions. Critically, we have shown that in BV-2 and EOC-20 cell lines express ErbB4 receptors, though we saw no expression of ErbB2 or B3 (Figure 1B). This discrepancy with the results of Dimayuga and colleagues (2003) might be attributed to the age at which the cells were harvested, the methods associated with harvesting and the morphology of microglia harvested. Microglia have two morphologies, amoeboid and ramified [31]. Amoeboid microglia are derived from blood born monocytes that migrate into the CNS [32,33]. These monocytes differentiate into an intermediate amoeboid microglia isoform that eventually matures into ramified microglia around P5 [32]. Amoeboid and ramified microglia often differ in their expression of receptors and phagocytotic activity [31,33]. The age at which N9 cells were originally harvested and the specific type of microglia cultured (amoeboid or ramified) might account for the differences shown here.

### NRG affects the expression of α7nAChR in microglia

At the NMJ NRG1 induced phosphotyroslyation is important in the up-regulation of AChRs at synapses. In microglia tyrosine phosphorylation is important for cell activation [34]. In our experiments, we specifically looked at the phosphotyrosylation of the ErbB4 receptors we found in the EOC and BV-2 microglia and the RAW 264.7 macrophage cell lines. In all three lines isolated, ErbB4 receptors exhibited robust phosphorylation in response to NRG1 addition to cultures (Figure 1B). In contrast, and not surprisingly given the lack of detectable ErbB2 and B3 expression, we saw no difference the phosphorylation states of these receptors when cells were treated with NRG1 (Figure 1B). Continuing, and in situations analogous to that at NMJs we were able to show that NRG1 addition can lead to an increase in α7nAChR in the BV-2 microglia cell line (Figure 2). Since ErbB4 appears to be the only NRG receptor present in BV-2 cells, we believe it may be forming homodimers to initiate a signaling cascade that results in the observed increases in α7nAChR expression. Interestingly, α7nAChR expression may be regulated by an Egr transcription factor [35] a family of transcription factors regulated by NRG [36,37].

### NRG affects TNF-α and IL-6 secretion in microglia

The cholinergic anti-inflammatory pathway in the periphery is a neuro-immune axis mediated by the vagus nerve [8]. The afferent arm of the vagus nerve detects elevated levels of endotoxins and pro-inflammatory cytokines that result from pathogen invasion and the activation of local macrophages. This signal initiates the synthesis of ACh by the efferent arm of the vagus nerve. ACh then presumably acts on macrophages via the α7nAChR, thereby decreasing TNF-α release [1,8,30,38]. A similar cholinergic system has been described in the brain with microglia [15]. Interestingly, NRG1 induces a 33% decrease in the secretion of TNF-α in microglia treated with NRG1, ACh and LPS, compared to only ACh and LPS (Figure 4A). In addition, NRG1 alone can induce a 76% decrease in IL-6 synthesis, another mediator of inflammation, in microglia challenged with LPS compared to LPS treated microglia alone (Figure 4B). We believe that this is the result of increased α7nAChR expression induced by NRG1 signaling through ErbB4 receptors (shown here in Figures 2 and 1 respectively). Our findings suggest that NRG1 signaling is antagonistic to the inflammatory response in microglia.

### Possible implications of NRG/ErbB4 signaling and the cholinergic anti-inflammatory pathway in the CNS and AD progression

Collectively these results suggest that NRG1 acting in concert with ACh may have a neuroprotective role in CNS. It has been suggested that inflammatory mechanisms are initiated by neurodegeneration in addition to inflammatory mediators. Inflammatory changes are evident in the overall AD brain and more specifically in the amyloid deposits that are rich in microglia [10]. Aβ peptides, resulting from the action of beta- and gamma-secretase proteases, play a major role in AD pathogenesis by promoting neurodegeneration by various mechanisms one of which includes activation of microglial cells. Aβ interacts with the LPS receptor CD14 [39], as well as other mediators, and stimulates pro-inflammatory pathways such as nuclear factor kappa B (NF-κB), which is necessary for cytokine production [40]. Several toxic Aβ peptides and the amyloid precursor protein (APP) are potent glial activators. Aβ proteins alone are capable of stimulating the NF-κB pathway. Activated microglial cells emitting inflammatory signals further recruit phagocytic cells, including astrocytes, to the site of Aβ deposition for the removal of damaged cells and cellular debris.

Overproduction of pro-inflammatory cytokines, including TNF-α, by microglia can significantly contribute to neuronal death. Wang and colleagues (2003) have shown that α7nAChRs present on tissue macrophages can mediate inflammation via a cholinergic system that inhibits inflammation by preventing the nuclear translocation of NF-κB [1]. Recent studies have further demonstrated that the brain also possesses a cholinergic anti-inflammatory pathway, similar to the one that exists in peripheral blood-borne macrophages [15]. Shytle and colleagues showed that acetylcholine and nicotine effectively attenuate the LPS-induced synthesis of TNF-α in microglial cells and as a result regulate microglial activation [15]. Cognitive impairment due to cholinergic deficiency is a major characteristic of AD. Interestingly, it has been reported that the loss of α7nAChRs enhances Aβ accumulation in early AD [29]. The activation of α7nAChR attenuates Aβ toxicity by a high affinity interaction with the Aβ peptide and as such retards the oligomerization of Aβ peptides [29]. Taken together these findings suggest a neuroprotective role for α7nAChRs.

NRG has been implicated in playing a neuroprotective role in the brain. In microglia it appears to have roles in cell survival, proliferation, migration and/or chemotaxis [26]. Previously, it has been implied that altered activity of NRG/ErbB signaling plays a role in AD pathogenesis [41] since ErbB4 has been reported to undergo presinilin-dependent gamma-secretase cleavage [42,43]. Loss of presenilin function, a protein involved in APP processing, leads to up-regulation of inflammatory markers. Although, its effect on microglial activation is still under scrutiny, presenilin is also involved in a variety of metabolic pathways and in the cleavage of transmembrane1 proteins, including ErbB4 [10,42,44]. However, increased concentration of microglia of plaques and sites of Aβ accumulation has also been associated with secretion of neuroprotective glia derived neurotrophic factor (GDNF), aside from simple phagocytosis, clearance and degradation of damaged tissue [45]. Activated microglia have been reported to be capable of expressing NRG1 [41,46,47] and the microglia in plaques demonstrate increased ErbB4 immunoreactivity [41], potentially as an inflammatory response to injury. Finally, it has been demonstrated that NRG plays a neuroprotective role following pro-inflammatory and stress gene expression due to focal ischemia [48,49]. Ischemic stroke results in brain injury and neuronal death as a result of inflammatory mediators. NRG can delay neuronal death by suppressing the expression of upwards of 30 ischemia-induced inflammatory and stress genes activated in microglia [48,49].

We suggest NRG signaling through the ErbB4 receptors may exhibit its neuroprotective role under inflammatory conditions by acting in concert with cholinergic anti-inflammatory pathway. Since the NRG/ErbB4 has always had close interactions with AChRs via PSD-95 [50] and other molecules, and both NRG and α7nAChRs have been previously identified to have neuroprotective roles, the collective effect of these molecules is not unreasonable and must be investigated further. In addition, the current evidence indicating both molecules are present in neuritic plaques and are important in the progression of AD emphasizes the importance of finding a cross-link between the two pathways as a potential therapeutic target for AD.
